# Trace fluorescent labeling for protein crystallization

**DOI:** 10.1107/S2053230X15008626

**Published:** 2015-06-27

**Authors:** Marc Pusey, Jorge Barcena, Michelle Morris, Anuj Singhal, Qunying Yuan, Joseph Ng

**Affiliations:** a iXpressGenes Inc., 601 Genome Way, Huntsville, AL 35810, USA

**Keywords:** trace fluorescence labeling, crystallization, screening, intensity

## Abstract

Fluorescence can be a powerful tool to aid in the crystallization of proteins. In the trace-labeling approach, the protein is covalently derivatized with a high-quantum-yield visible-wavelength fluorescent probe. The final probe concentration typically labels ≤0.20% of the protein molecules, which has been shown to not affect the crystal nucleation or diffraction quality. The labeled protein is then used in a plate-screening experiment in the usual manner. As the most densely packed state of the protein is the crystalline form, then crystals show as the brightest objects in the well under fluorescent illumination. A study has been carried out on the effects of trace fluorescent labeling on the screening results obtained compared with nonlabeled protein, and it was found that considering the stochastic nature of the crystal nucleation process the presence of the probe did not affect the outcomes obtained. Other effects are realised when using fluorescence. Crystals are clearly seen even when buried in precipitate. This approach also finds ‘hidden’ leads, in the form of bright spots, with ∼30% of the leads found being optimized to crystals in a single-pass optimization trial. The use of visible fluorescence also enables the selection of colors that bypass interfering substances, and the screening materials do not have to be UV-transparent.

## Abstract 2

The presence of a covalently bound fluorescent probe at a concentration of <0.5% does not affect the outcome of macromolecule crystallization screening experiments. Additionally, the fluorescence can be used to determine new, not immediately apparent, lead crystallization conditions.

## 1. Introduction   

The purpose of macromolecule crystallization screening experiments is the identification of lead conditions for the growth of crystals that are ultimately suitable for X-ray diffraction analysis. Although it has been calculated that only ∼300 experiments are needed to obtain crystals if a protein has a 2% chance of crystallizing (Segelke, 2001 ▸), in many instances considerably more experiments are carried out in an effort to increase the chances of finding the ever-elusive lead condition. The volumes of the experimental tests are often reduced to maximize the number of trials that are carried out with a limited amount of protein. The generation of large numbers of crystallization plates leads to robotics for setting them up and for reviewing the results. Compounding these issues is the fact that crystallization screening experiments are typically carried out as a yes/no process: yes, there are crystals or no, there are not. Outcomes that do not immediately present as crystals are often dropped from further consideration, even though they may be lead crystallization conditions (Pusey *et al.*, 2008 ▸).

Fluorescence is a powerful method for the identification of macromolecule crystals. A guiding paradigm for its use is that intensity, which is a function of the local density of the fluorescing species, is equal to structure, as the most densely packed form of a protein is the crystalline form. Three approaches have been presented for using fluorescence: (i) the use of intrinsic protein UV fluorescence (Judge *et al.*, 2005 ▸), (ii) the addition of a probe to the protein that only fluoresces when bound in a hydrophobic environment (Groves *et al.*, 2007 ▸) and (iii) the covalent labeling of the protein with a trace amount (typically ∼0.2%) of a visibly fluorescing probe (Forsythe *et al.*, 2006 ▸). Several instruments are commercially available for the UV imaging of protein crystallization plates. To use UV fluorescence, the protein must contain tryptophan residues. For the added and trace-labeling approaches standard laboratory epifluorescence microscopes can be used with direct viewing of the results. In all cases a review of the plate results can be rapidly carried out by simply looking for intensity rather than straight lines.

We have implemented and further developed the trace fluorescent labeling (TFL) approach as an aid in identifying macromolecule crystallization conditions (Forsythe *et al.*, 2006 ▸). The method involves covalently labeling from 0.1 to 0.5% of the protein molecules with a visible fluorescent probe, usually on a side-chain amine. Upon crystal formation the fluorescent label is concentrated, producing a bright fluorescence that is readily detected by visual examination. Fluorescence from amorphous precipitate, which is not as concentrated, is not as intense, and in fact crystals buried in or under amorphous precipitate are easily spotted. We have previously shown that the trace-labeling method does not affect the nucleation rate at bound probe levels of <1% and that the X-ray diffraction data are not affected at probe concentrations of up to 5–10% (Forsythe *et al.*, 2006 ▸). However, the previous study focused on known crystallization conditions for well characterized model proteins. Here, we present an extended study to directly compare the effects of trace-labeled *versus* nonlabeled protein using a 96-condition screen.

## 2. Materials and methods   

Many of the proteins used in this study, including PCNA and PCP (see Table 1 ▸), were cloned from the hyperthermophile *Thermococcus thioreducens* (Pikuta *et al.*, 2007 ▸; Hughes & Ng, 2007 ▸). Unless otherwise noted, the standard purification was a 348 K heat cut of the lysed cell supernatant for 30 min and ion-exchange chromatography on a low-pressure QAE column (Bio-Rad), followed by gel filtration using a 1.5 × 75 cm S200 column (GE) equilibrated in 0.05 *M* Na HEPES, 0.1 *M* NaCl pH 7.5, which also served as the standard crystallization buffer. Proteins were quantified using their absorptivities as calculated on the basis of their amino-acid content (Gasteiger *et al.*, 2005 ▸).

### 2.1. Proteins   

Phaseolin (Phas) was purified from kidney beans obtained from a local grocery store using the method of Suzuki *et al.* (1986 ▸). PCNA from *T. thioreducens* was purified by the method of Byrne-Steele & Ng (2009 ▸). The gene for *Haemophilus influenzae* IPPase was assembled from oligonucleotides using previously described methods (Marsic *et al.*, 2008 ▸). The synthesized gene was inserted between the NdeI and BamHI sites of pET-3a (Novagen, USA) through homologous recombination *in vivo*. To facilitate protein purification, a His_6_ tag (MHHHHHHQ) was added to the N-terminus of the protein. The plasmid was propagated in *Escherichia coli* strain DH5α (Genlantis Inc., San Diego, California, USA). Error-free clones were selected and were subsequently transformed into *E. coli* Rosetta strains (Genlantis Inc., California, USA) for protein production. A 100 ml overnight starter culture was resuspended in 4 l LB medium in the presence of carbenicillin and chloramphenicol and was further cultured at 30°C. Recombinant protein production was induced with 0.5 m*M* IPTG when the optical density at 600 nm reached 0.6 and induction was maintained for 18–20 h at 18°C with vigorous shaking at 250 rev min^−1^. Cells were harvested by centrifugation and cell pellets were kept at −80°C until use. Protein purification after cell lysis was carried out by Ni^2+^-affinity chromatography followed by size-exclusion chromatography. The proteins transferrin (Sigma, catalog No. T4132) and glucose oxidase (Sigma, catalog No. G7141) were dissolved in the standard crystallization buffer and used without any further purification. Bovine serum albumin (BSA; Sigma, catalog No. A7030) was dissolved in 0.05 *M* sodium borate buffer pH 8.75 at ∼100 mg ml^−1^ and then divided into two pools. The first was directly chromatographed by size-exclusion chromatography on a 1.5 × 100 cm S100 column (GE Healthcare) equilibrated in 0.05 *M* HEPES, 0.1 *M* NaCl pH 7.5. The second was trace fluorescently labeled using 0.1 ml reactive probe stock solution for derivatization per millilitre of stock protein solution. After 15 min reaction time, 50 µl 0.1 *M* glycine was added, the reaction mixture was added back to the stock solution and the mixture was then passed through the S100 column. The monomeric fractions were determined using SDS–PAGE, pooled, concentrated to 100 mg ml^−1^ by ultrafiltration and diluted to 50 mg ml^−1^ for crystallization trials.

### 2.2. Trace fluorescent labeling (TFL)   

Proteins were trace fluorescently labeled with either carboxyrhodamine-succinimidyl ester (CR; Invitrogen, catalog No. C6157) or Texas Red (TR; Invitrogen, catalog No. T6134). The procedure used evolved from one previously described (Pusey *et al.*, 2008 ▸). Stock probe solution was prepared by dissolving the contents of the bottle *in situ* with 1 ml anhydrous dimethylformamide. The stock probe solution was stored at 253 K. Briefly, the protein was concentrated to >15 mg ml^−1^ and an 800 µl solution was prepared containing 12 mg (*i.e.* at 15 mg ml^−1^ concentration) aliquoted as the stock protein solution. From this, 80 µl were withdrawn and centrifugally buffer-exchanged using a 0.5 ml column (Pierce) into 0.05 *M* borate pH 8.75. To the buffer-exchanged protein was added, with mixing, 0.6 µl stock probe solution, with the bottle warmed to room temperature prior to being opened. The reaction mixture was set aside while the centrifugal buffer-exchange column was re-equilibrated in crystallization buffer, after which the mixture was passed through to both carry out the buffer exchange and remove the unbound probe. The eluted protein was added back to the stock protein solution and the volume was adjusted to 1.0 ml with crystallization buffer.

### 2.3. Crystallization screening   

Two protein solutions were prepared from the stock protein solution: one trace-labeled as described above and a second with the same final protein concentration that was not trace-labeled. Crystallization screens were set up using Crystal Screen HT (Hampton Research, catalog No. HR2-130). Three sitting-drop plates of each protein preparation, labeled and not labeled, were prepared using a Nanodrop robot in Corning CrystalEX plates (Hampton Research, catalog No. HR8-140) using 35 µl reservoir solution and protein:precipitant volumes of 400:400, 400:200 and 800:200 nl. The nonlabeled protein plates were set up first followed by the labeled protein plates, and all plates were set up in a single session. Immediately upon completion of the dispensing operations each plate was sealed with clear film and then placed into an incubator at 18°C.

### 2.4. Fluorescent imaging   

Plates were originally imaged using a visible scanning fluorescence microscope assembled in-house. The system consists of an *XY* stage to move a tray holding the plate. The microscope system is a basic epifluorescence system using a high-intensity 530 nm light-emitting diode (LED; Mightex Systems) for illumination, which passes through a low-pass excitation filter (Omega, XF1080), is reflected to the sample using a dichroic mirror (Omega, XF2012) focused on the sample with a 5× objective, which also serves to collect the emitted fluorescence, which passes through the dichroic mirror and then through a high-pass emission filter (Omega, XF3021) and is subsequently focused onto a camera (IDS Imaging, UI-5580-SE) by an achromatic lens pair (Edmund Optics). Removal of the filter cube and placing a white-light source below the plate enabled transmission imaging. The plate-imaging operations were controlled by a PC using software written in-house in C++. It took ∼30 min to acquire the images from the Corning plates, with the scanning times scaling proportionately with the number of drops per precipitant condition. Plates were scanned within 4 h of being set up and then daily for the next 3 d, followed by weekly for the next two months. Later plates were scanned using a commercial version (Crystal X2, iXpressGenes/Molecular Dimensions) of the above-described microscopy system. The major differences are that the latter instrument can image at two different fluorescent wavelengths and with white light without having to change the filters or source illumination and the scanning time for a single color is ∼14 min.

### 2.5. Plate and image analysis   

Recorded images were manually reviewed and the presence or absence of crystals was determined by the fluorescence intensity (Forsythe *et al.*, 2006 ▸). For the final scoring image analysis the screening plates were reviewed manually by low-power transmission microscopy at the end of eight weeks. Scores for the nonlabeled and labeled solution plates were recorded for each well (three per precipitant condition). Following visual examination the final recorded fluorescent images were reviewed and the fluorescence scores were adjusted accordingly. This last review step served to remove any salt crystals or other artifacts from the scores owing to their lack of fluorescence and to identify ‘bright-spot’ or other features which were otherwise not identifiable in the white-light images. Using a modified version of the scoring scale accompanying the screen solutions, the ‘bright-spot’ outcome was assigned a score of 4. Scores of >4 were for results that were identifiable under white light and indicated some form of structure. These included three-dimensional crystals (scores of 8 or 9), two-dimensional plates (7), needles (6) and ‘urchins’, dendrites and spheroids (5); essentially, any outcome that showed high fluorescence intensity and that when viewed under white light could be taken as a basis for subsequent optimization trials. Precipitates with indeterminate bright regions were those that on white-light viewing would be dismissed as precipitate but which showed bright regions that could not be ascribed to any observable structure under fluorescent illumination. This category was the primary source of lead conditions.

### 2.6. Optimization trials   

Optimization of lead conditions was carried out using capillary counter-diffusion (CCD; García-Ruiz, 2003 ▸; Ng *et al.*, 2003 ▸) using an approach developed in-house. CCD optimization solutions were prepared at four ratios of the initial stock crystallization solution. For a crystallization solution consisting of precipitants *A* and *B* with buffer, the four solutions were (1) 100% each of *A* and *B*, (2) 50% *A* and 100% *B*, (3) 100% *A* and 50% *B*, and (4) 50% each of *A* and *B*, with buffer at 100% for all solutions. If no buffer is in the original screen solution then 0.1 *M* HEPES pH 7.5 was used. If only one precipitant was present then the four optimization solutions consisted of 100, 75, 50 and 25% of that component. Initially, CCD optimizations used polycarbonate tubing with internal and outer diameters of 200 and 250 µm, respectively (Paradigm Optics). This was latterly changed to CCD optimizations using 4 cm lengths of borosilicate glass tubing with internal diameters of 0.3, 0.2 or 0.1 mm (VitroCom). When using polycarbonate tubing the number of capillaries to be set up was determined, and an average length per capillary of 3 cm was assumed. A length of tubing to accommodate all these capillaries was cut from the reel of tubing. One end was affixed to a microsyringe and the other was left free. The free end was placed into the protein solution at 30 mg ml^−1^ and the protein was aspirated into the tube. When the liquid meniscus was observed close to the syringe the plunger was pushed to provide slight positive pressure, such that a droplet of protein solution slowly formed at the free end of the capillary. The free end was then sealed by jabbing it into a shallow container of soft wax, the tubing was cut ∼3 cm from the end and the new free end of the long capillary length was again sealed with wax. The short length of protein-filled tubing was inserted open end down into the optimization solution and the tube was capped. Alternatively, 4 cm lengths of glass capillary tubing were filled by capillary action from the stock protein reservoir, the nonfilling end was sealed with soft wax and the tube was inserted into the optimization solution open end down. The reservoir solution was 40 µl precipitant in 1.2 ml titre tubes (E&K Scientific, catalog No. 684510-R). The tubes were closed using caps (E&K Scientific, catalog No. 64108-P). An advantage of this approach is that several capillaries can be placed within a single tube while maintaining a high precipitant:protein ratio. Optimizations were also carried out in some instances using Crystal Former CCD plates (Microlytic/Anatrace) or sitting-drop plates. In two of the instances reported here (PCP and HiIPP) optimization experiments were carried out with sitting-drop plates using the stock crystallization solutions at 90% concentration and made 0.1 *M* in ionic liquid (Pusey *et al.*, 2007 ▸) by dilution of a 1 *M* stock ionic liquid solution.

## 3. Results   

### 3.1. Labeling of the protein   

**Figure 1:**
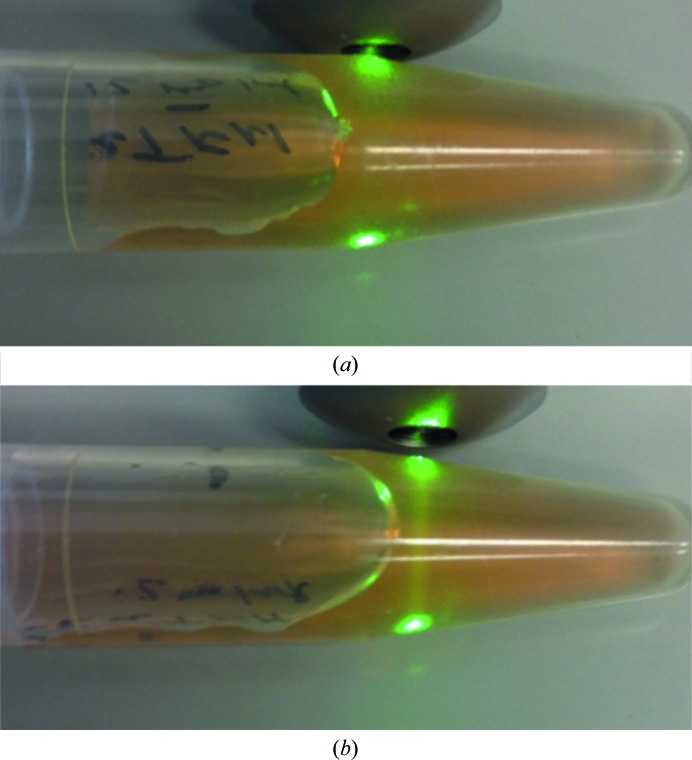
Using a green laser pointer to verify that protein has been trace fluorescently labeled. The protein is bovine apo transferrin at 12 mg ml^−1^. (*a*) Unlabeled protein; (*b*) labeled protein. Note the emission from the laser in (*b*) which is not present in (*a*).

The protein-labeling process typically took ∼10–15 min. Shorter times could be achieved if two desalting columns were simultaneously prepared in reaction and crystallization buffer, respectively, with the first used immediately after the second. If several proteins are to be labeled the process can be carried out in parallel. The reactive fluorescent probe was either added directly from the (pre-warmed) bottle or was pre-aliquoted into PCR tubes, which were then stored at −20°C prior to use. After labeling, the final protein solution is at best very faintly colored. A check of the labeling process can be made by shining a green laser pointer (excitation wavelength 530 nm) through the solution, as shown in Fig. 1 ▸. Here, one can clearly see the fluorescence owing to the probe in the labeled, but not the unlabeled, solution.

The procedure given is to prepare 1 ml protein solution at the desired final crystallization screening concentration. Smaller volumes can be accommodated by varying this procedure. Empirically, the two centrifugal desalting steps add ∼50 µl to the protein solution being labeled. One can buffer-exchange smaller volumes, but an additional stacker volume of buffer needs to be added (Pierce/Thermo instruction sheet for catalog No. 89882). Assuming that the sample plus stacker gives a starting volume of 80 µl and that the volume after the second buffer exchange will be ∼130 µl, then reducing the stock protein volume by this amount prior to removing a tenth of the volume to be derivatized should be sufficient for labeling volumes of less than 1.0 ml. For volumes in the <100–300 µl range smaller centrifugal desalting columns can be employed.

Fig. 1 ▸ also introduces an important point about the TFL process. The goal is to only label ∼0.1–0.2% of the protein or 1–2 protein molecules per thousand. Heavier labeling is counterproductive, giving rise to higher background fluorescence upon imaging. The labeling procedure given was empirically determined to quickly achieve this goal while avoiding the introduction of concentration and quantification steps that would slow the process down.

### 3.2. Crystallization screening results   

**Figure 2:**
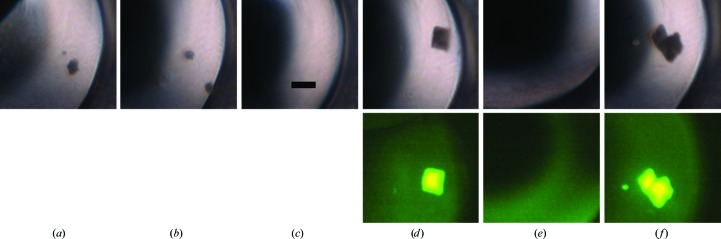
Variation in crystallization outcomes owing to the stochastic nature of crystal nucleation. Shown is well B3a (protein:precipitant ratio of 1:1) for six plates set up for protein Tt186. (*a*), (*b*) and (*c*), unlabeled protein; (*d*), (*e*) and (*f*), TFL protein. The corresponding fluorescence images are shown below in (*d*), (*e*) and (*f*). All images are on the same scale, with the bar shown in (*c*) corresponding to 200 µm.

**Figure 3:**
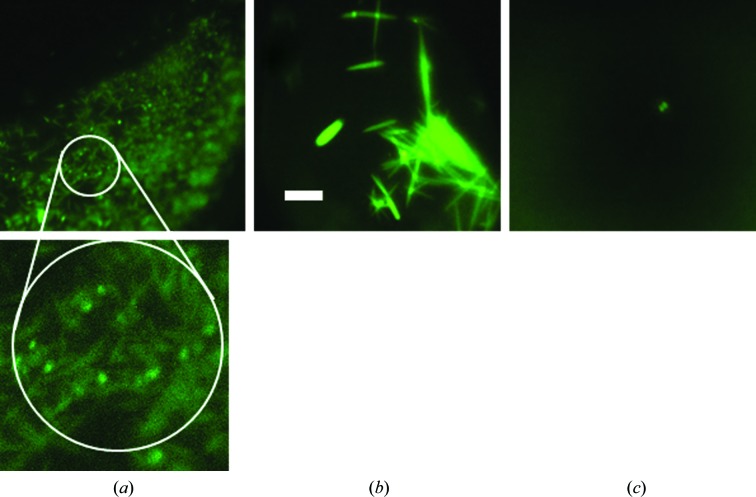
Variation in crystal scores for a single condition in a single plate. Protein Tt94, condition D7a (protein:precipitant ratio of 1:1). (*a*) Scored as 6 (needles); (*b*) scored as 8 [three-dimensional crystal(s)]; (*c*) scored as 4 (‘bright spots’). All images are on the same scale, with the scale bar in (*b*) corresponding to 200 µm. The indicated region of (*a*) is enlarged, showing the needles. Note that they show a light-pipe effect, with the ends glowing brighter than the body of the needles.

Crystal nucleation is a stochastic process. A previous report (Newman *et al.*, 2007 ▸) has shown that one does not always obtain the same crystallization results from the same screen and protein. Accordingly, for this comparison test we set up three plates of labeled protein and three of unlabeled protein, all from the same preparation, for our screens. For a given protein, conditions that gave crystals in one plate often did not always give crystals in the corresponding well of another, independent of the presence or absence of a bound fluorescent probe, as shown in Fig. 2 ▸. Additionally, we used three ratios of protein to precipitant for each screen condition, and again the results were not always consistent for all plates. For any one 96-condition screen there are 288 possible crystallization wells. For simplicity, we report the outcomes on the basis of the conditions, not on the basis of the wells. However, each well was independently scored. Thus, the same screen condition, depending upon the plate and/or well, may result in a clear solution (score = 1), phase separation (score = 2), precipitate (score = 3), bright spots (score = 4), urchins, spheroids, dendrites *etc.* (score = 5), needles (score = 6), two-dimensional plates (score = 7) and three-dimensional crystals (scores of 8 or 9). An example of this is shown in Fig. 3 ▸. Note that in Fig. 3 ▸(*b*) both one-dimensional (needle) and three-dimensional crystals are present. The score for the well is that of the highest scoring outcome, in this case three-dimensional crystals.

The results from the proteins tested fell into two categories; those which resulted in a number of crystallization hits (facile crystallizers) upon screening and those which did not. A cutoff point for division into one or the other category was arbitrarily set at three separate crystallization conditions which resulted in at least one well with a score of 8 or higher. As there are three plates, with three wells per condition, there are nine possible wells for crystallization for any screen condition for both the labeled and unlabeled protein trials. In general, no further trials were conducted on those proteins having >3 crystallization conditions for both the labeled and unlabeled protein, although this cutoff point was disregarded for the proteins PCP and HiIPP. Lead conditions were those outcomes where one or more wells out of the nine labeled had a score of 4 (‘bright spots’), but none of the other wells, labeled or unlabeled, for that condition had a score of ≥5.

A total of 22 proteins were tested. Of these, 16 were derived from the hyperthermophilic archaeon *T. thermoreducens* (Pikuta *et al.*, 2007 ▸) and six from other sources. Table 1 ▸ summarizes the data obtained. The data in the columns for the scores of 5 through 8–9 give the number of conditions with these scores for the labeled protein (numerator) and the unlabeled protein (denominator). Note that for many conditions where there were one or more well scores of 4 there were other wells with a score of ≥5. The ‘unique leads’ column shows the number of conditions where there was a score of 4 with none of the other wells for that condition having a score of ≥5, and the ‘leads tested’ column the number of those from the ‘unique leads’ column that were subjected to optimization screening. The selection of conditions for subsequent optimization testing was only carried out on the basis of the fluorescence image score, *i.e.* the trace-labeled protein. These were all lead conditions that were not evident on the basis of white-light observations.

The data in Table 1 ▸ demonstrate the effects of the presence or absence of trace labeling on the crystallization outcome. From the totals in the bottom row we see that overall there were consistently more labeled than unlabeled outcomes giving the indicated scores, although there are several instances evident where for a specific protein and outcome the unlabeled results were better than the labeled results. There is only one instance where the unlabeled protein gave a structured outcome but the labeled did not: holo-transferrin, with a score of 7. While these results may be taken to indicate that labeling positively affects crystal nucleation, we interpret them to indicate the improved facility in observing the outcome by reason of using fluorescence.

### 3.3. Basis for ‘bright spots’ as a lead condition   

The data provide an experimentally derived basis for the use of ‘bright spots’, with a score of 4, for the determination of lead conditions. The discovery of the utility of these outcomes as leads was initially fortuitous (Pusey *et al.*, 2007 ▸) based upon the intensity = structure paradigm that forms the basis for using fluorescence for crystal detection. Data that further supports this are shown in Table 2 ▸, which shows the number of screen conditions for proteins that had a score of 4 and the numbers of these conditions where wells also had scores of between 5 and 9 for 12 of the proteins used. The data are shown on the basis of screen conditions. There are three plates for each crystallization condition, with each condition having three wells at different ratios of protein to precipitant. Thus, there are nine independent experiments over three different ratios of protein:precipitant for each screen condition for labeled and unlabeled protein. For a given condition it is possible to have three different scores for the three wells, as shown in Fig. 3 ▸. However, the score for the condition would be the highest of the three, in this case 8. For lead-optimization purposes, only those conditions where a score of 4 was the highest for that condition over all six plates were considered to be unique and tested (Table 1 ▸).

**Figure 4:**
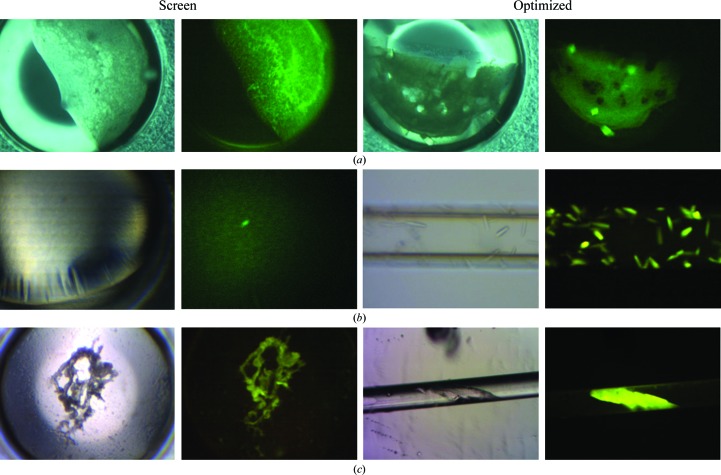
Fluorescent bright spots as crystallization lead conditions. The pairs of images to the left are the transmission (white light) and fluorescent images of the initial crystallization screen. The images to the left are the transmission and fluorescent images of the optimized outcomes. (*a*) HiIPPase, lead Hampton Research Crystal Screen HT (HRHT) condition E1, optimization with 90% E1 + 0.1 *M* 1-ethyl-3-methylimidazolium chloride. (*b*) Tt169, lead HRHT condition D6, optimization using Crystal Former HT plates (Microlytic/Anatrace). The capillary width is 100 µm and the imaged volume is ∼15 nl. (*c*) Tt97, lead HRHT condition C2; the capillary in (*c*) has an internal diameter of 200 µm.

The nature of the features giving ‘bright spots’ is unclear at present. From the fluorescence intensity we assume them to be owing to densely packed, likely microcrystalline, protein. However, the inferred crystalline nature has not been confirmed by diffraction analysis. One possibility is that they are nucleation events that have been overtaken or poisoned by amorphous precipitation, although they are not always associated with precipitated protein. The score of 4 is only assigned on the basis of a review of the fluorescent images after manual scoring, which means that these are results which would not have been taken as lead conditions on the basis of normal plate observations. Wells given this score have generally been scored as a 3 (precipitate) or in some instances a 1 or 2 (clear well or phase change) under white light. Fig. 4 ▸ shows several examples of ‘bright-spot’ conditions that were optimized to crystals and also gives an indication of the variability in what is observed under white light.

Optimizations were carried out using capillary counter-diffusion, as this has been shown to typically have a higher success rate than ‘standard’ vapor-diffusion methods (Ng *et al.*, 2003 ▸). CCD optimizations were usually made using a homebrew approach, with the capillary either being glass or polycarbonate. Visible fluorescence observations could be made using either material.

While assignment of a score of 4 is at present somewhat subjective, ∼30% of the conditions assigned this score have been optimized to crystalline outcomes. This reduces to ∼24.5% if we remove the facile crystallizer PCP from the data. As these are results that would otherwise likely have been discarded as nonproductive outcomes on the basis of how they presented under white-light observation, they represent a significant further increase in the success rate obtained from using the TFL approach. It is likely that a second and third round of optimization trials for these conditions would further increase these success rates. Only one protein, BSA, did not give crystals upon optimization of the bright-spot conditions. Three other proteins which did not have three-dimensional crystals over the six plates in the initial screening round, Tt46, Tt82 and Tt106, did give crystals upon optimization of the bright-spot lead conditions.

## 4. Discussion   

The purpose of crystallization screening trials is to identify lead crystallization conditions. TFL is put forth as a means for rapidly identifying crystals and, more critically, potential lead crystallization conditions from screening trials. It is important here to point out the obvious that once lead conditions have been identified then one has the desired information and the subsequent optimization of lead conditions and growth of crystals for diffraction studies can be carried out using protein that is not labeled. Although it has previously been shown that the presence of a covalently bound label does not adversely affect the quality of the data obtained (Forsythe *et al.*, 2006 ▸), by this considerations of the effects of the bound probe on the diffraction data are rendered moot. The only remaining concern is what effect the presence of the bound probe has on the screening results.

The data presented above clearly indicate that when followed over a range of proteins the TFL approach does not affect the outcome of the screening results. In fact, an optimistic interpretation of the results shown in Table 1 ▸ could be that the presence of the label gives the appearance of improving the nucleation rate. That the outcomes for the labeled and unlabeled protein do not always exactly match is not surprising. Crystal nucleation is a stochastic process, and it is commonly found that setting up multiple instances of a given condition does not always result in the same outcome (Newman *et al.*, 2007 ▸). However, the numbers of hits for scores of ≥5 over all of the experiments is consistently greater for the TFL protein. We attribute this to the greater ease by which the presence of the label enables the identification of outcomes, and not to a positive effect of the bound probe on the crystal-nucleation process. We also point out that there is considerable variation from protein to protein in the numbers presented in Table 1 ▸, and that the totals are just that: the sum of results over 22 different proteins. In some instances only one crystal was found over all six plates. The scoring process itself was initially carried out manually using a transmission microscope. It was only after this that the scores for the labeled proteins were adjusted by reference to the fluorescent images. The TFL images only impacted the nonlabeled images when structures identified as salt (*i.e.* that did not fluoresce) were found for the same conditions for both the labeled and unlabeled protein.

An advantage of the TFL approach is that it can be carried out using lower-cost visible optical systems and the wavelengths employed can be selected to avoid potentially interfering substances. Also, by using a LED instead of a laser for illumination one can directly view the results through the microscope without resorting to a camera for imaging, which is especially not recommended when using UV illumination. This approach has previously been shown to work well with a range of model proteins, with minimal effects on the quality of the diffraction data obtained at bound probe levels as high as 10% (Forsythe *et al.*, 2006 ▸). Here, we have now tested it with a wider range of proteins with a more extensive exploration of finding hidden leads.

The use of visible fluorescence also negates considerations about the UV transmission of crystallization plates and sealing films. Specialized UV transmissive optics for the microscopy system are not required, and the imaging camera itself, if employed, need only be suitable for visible wavelengths. This reduced concern for materials is shown in Fig. 4 ▸(*c*), where a crystal inside a polycarbonate tube was imaged inside a semi-opaque polypropylene tube.

A major advantage of the fluorescence approach stems from being able to derive additional, not otherwise obvious, lead conditions from a crystal screening plate. It was found that ∼30% of these conditions could yield crystals in a first-pass optimization screen. The success rate is based on the number of conditions that were tested and thus represents multiple successes for most proteins. The rate would be ∼89% if it were determined based upon the number of proteins that were optimized based upon the TFL-derived leads. Except for the protein PCP, only those proteins having ≤3 conditions giving crystals over the six plates were optimized. This was to minimize the impact that the inclusion of the ready crystallizers would have on the success rate, as shown by the effect of the PCP results. In this work, the identified lead conditions were empirically selected on a broad basis to capture as many leads as possible. An indication of the range of white-light outcomes that gave rise to a score of 4 is shown in Fig. 4 ▸, and so far no correlation has been found that would indicate, on the basis of the white-light image alone, what might be a potential lead condition as shown by the presence of ‘bright spots’ in the fluorescence image. Sufficient additional experiments may eventually give a basis for better identifying these conditions. However, an increase in the number of hits, particularly in those instances where there previously were none, is judged to be worth an ‘only’ 25–30% success rate in lead screening.

A novelty of the fluorescence approach is apparent in the data. In Fig. 3 ▸(*a*) we see a light-pipe effect from the needle-shaped crystals, which manifests as bright spots under fluorescent illumination. On occasion needles are mixed in with amorphous precipitate, and as a result bright-spot outcomes occasion even closer scrutiny of the results using white-light microscopy. This light-pipe effect is also found with rod-shaped crystals. Another benefit is shown in the optimized outcomes in Fig. 4 ▸(*a*). While the two crystals on the edges are clearly visible with white-light illumination, the fluorescent image shows that at least four crystals are present, two of which are buried under the precipitate and are not visible under white-light illumination.

Overall, the presence of a covalently attached fluorescent probe is shown to not have a negative impact on the crystallization screening outcome. On the contrary, we find powerful advantages to using fluorescence, among them that the screening results can be surveyed considerably more quickly, with more leads identified, when using intensity as a search feature as opposed to straight lines. Additional benefits come from being able to identify potential lead conditions that otherwise would have been discarded based upon transmission microscopy observations. The TFL procedure can be rapidly carried out and does not represent a serious interruption of the workflow prior to setting up a screening plate.

## Figures and Tables

**Table 1:** Summary of labeled *versus* unlabeled plate data NT, not tested; NI, not included.

	Score of labeled/unlabeled							
ID	4	5	6	7	89	Unique leads	Leads tested	Testedcrystals
Tt46: ssDNA-specific exonuclease	31	13/6	1/1	1/0	0/0	22	22	7
Tt55: translation initiation factor	31	26/21	12/14	3/1	3/3	14	14	4
Tt71: intracellular protease	13	5/5	23/26	0/0	7/8	5	NT	NT
Tt75: prolyl endopeptidase	29	18/13	3/0	5/4	1/5	15	11	1
Tt80: HAD-family sugar phosphatase	31	17/12	1/0	0/0	9/6	24	NT	NT
Tt81: haloacid dehalogenase	11	42/54	9/5	0/0	18/16	0	NT	NT
Tt82: HAD-superfamily hydrolase	23	5/8	1/0	5/4	0/0	22	12	6
Tt94: RNA 3-terminal phosphate cyclase	21	9/8	11/11	0/0	8/8	14	NT	NT
Tt97: aspartate racemase	14	24/2	3/2	0/0	6/5	8	NT	NT
Tt102: endonuclease methyltransferase	20	9/6	2/4	1/0	1/1	15	15	1
Tt106: nucleotide kinase	16	5/3	0/0	0/0	0/0	15	14	4
Tt141: inorganic pyrophosphatase	11	21/23	13/14	4/6	20/12	3	1	1
Tt186: alcohol dehydrogenase	10	18/21	10/8	4/5	15/12	3	NT	NT
Tt189: nucleoside diphosphate kinase	21	10/10	1/1	1/1	7/3	3	NT	NT
PCP: pyroglutamate aminopeptidase	42	36/22	32/8	4/2	8/2	20	20	15
PCNA: proliferating cell nuclear antigen	21	22/22	10/6	3/0	14/11	8	NT	NT
Phas: kidney bean phaseolin	13	17/19	0/0	0/0	15/17	5	NT	NT
HiIPP: *Haemophilus influenzae* IPPase	47	24/25	1/0	0/0	16/20	11	NI	NI
hTFN: holo transferrin	13	4/6	0/0	0/2	2/2	13	NT	NT
BSA: bovine serum albumin	10	2/2	1/1	1/0	0/0	9	9	0
GOL: glucose oxidase	13	10/7	3/2	0/0	6/6	6	10	NT
								
Totals		337/295	137/103	32/25	156/139		128	39

**Table 2:** Association of ‘bright-spot’ conditions with outcomes having a higher score

	Conditions with a score of	That also had wells with a score of			
Protein	4	5	6	7	8 or 9
Tt46	31	10	0	0	0
Tt55	31	18	3	3	2
Tt71	13	5	8	0	1
Tt75	29	13	1	2	1
Tt82	23	2	0	0	0
Tt94	21	5	6	0	3
Tt97	14	6	1	0	0
Tt102	20	3	2	1	1
Tt106	16	2	0	0	0
Tt141	11	7	2	2	7
Tt186	10	5	3	1	3
Tt189	21	9	1	2	3
